# A Novel ALDH2 Inhibitor for the Treatment of Alcohol Use Disorder: Preclinical Findings

**DOI:** 10.3390/cells15020123

**Published:** 2026-01-09

**Authors:** Randall D. Marshall, Andrew Fowlie, Adam Sabouni

**Affiliations:** Sophrosyne Pharmaceuticals Inc., 540 W. Madison Street, Suite 2500, Chicago, IL 60661, USA; afowlie@sophrosynepharma.com (A.F.); asabouni@sophrosynepharma.com (A.S.)

**Keywords:** sophrosyne, SOPH-110S, ALDH2 inhibitor, disulfiram, alcohol use disorder, AUD, DETC-MeSO, alcohol, ethanol

## Abstract

**Highlights:**

**What are the main findings?**

- SOPH-110S is a novel ALDH2 inhibitor intended to be developed as a long-acting injectable for the treatment of alcohol use disorder
- Preclinical studies that supported an approved IND in the US demonstrated high potency, acceptable safety, no off-target effects in a panel of 84 common targets, no CYP interactions, and positive proof-of-mechanism data in rats.

**What are the implications of the main findings?**

- SOPH-110S has preclinical promise supporting clinical evaluation.
- SOPH-110S data, to date, support its potential as a safer, better-tolerated, long-acting injectable therapy for AUD that is superior to oral disulfiram.

**Abstract:**

Background: Alcohol use disorder is a common condition with high morbidity and mortality and no highly effective treatments. Achieving and maintaining abstinence is necessary or desired for many persons with AUD, but is difficult due to the nature of the condition. Pharmacologic inhibition of the enzyme ALDH2, which increases levels of the substrate acetaldehyde when alcohol is imbibed, can serve as a powerful enforcer of efforts to remain abstinent. Disulfiram is an approved ALDH2 inhibitor via its active metabolite DETC-MeSO, but has many limitations, including numerous adverse effects, hepatotoxicity, oral administration, and unpredictable mechanistic activity. Methods: SOPH-110S, an analog of DETC-MeSO, was evaluated in a series of experiments to assess mechanism, pharmacokinetics in male beagle dogs, cardiovascular safety in telemeterized male beagle dogs, selectivity, off-target activity, CYP inhibition, and proof of mechanism in a rat model that included dosing and alcohol challenge followed by analysis of liver ALDH2 inhibition. Results: SOPH-110S showed high potency with a comparable IC_50_ vs. positive controls and no physiologically relevant off-target binding in an 84-target panel. It did not inhibit or induce any major CYP enzymes or meaningfully inhibit the hERG channel. After 10 days’ dosing in rats, followed by administration of alcohol, SOPH-110S was a highly potent, dose-dependent inhibitor of ALDH2, comparable to DETC-MeSO. No cardiovascular safety concerns were found at multiples above expected clinical doses. Conclusions: The preclinical data support further clinical study of SOPH-110S as a potential ALDH2 inhibitor treatment for AUD. The FDA approved the IND to conduct a first-in-man phase 1 study in September 2025.

## 1. Introduction

Alcohol use disorder (AUD) is a chronic disorder characterized by an impaired ability to stop or control alcohol use despite adverse social, occupational, or health consequences, with an estimated incidence of 3.7% of adults globally. AUD was responsible for an estimated 2.6 million deaths globally in 2019, and for 13% of all deaths among persons 20–39 years of age [1].

It is the most common substance use disorder in the US, with an estimated prevalence in 2022 of 28.1 million adults (10.9% in the 18-year and older age group) and 757,000 adolescents (2.9% in the youth ages 12 to 17 age group) [2]. In US public facilities alone, which only represent about half of all treatment facilities (17,561/28,900), 440,540 individuals received inpatient treatment for AUD in 2022 [2,3]. Thus, it is estimated that nearly 900,000 patients per year receive inpatient treatment for AUD in the US. Based on clinical practice, it is expected that the majority are discharged with the explicit goal of long-term abstinence.

The relative risk for mortality increases across the spectrum of daily alcohol intake, beginning from low-volume drinking to heavy drinking [4]. Chronic heavy drinking is also associated with increased risk for cancers, pancreatitis, and the spectrum of alcohol-related liver disease, which includes steatosis, fibrosis, alcohol-associated hepatitis, and cirrhosis [5]. It is estimated that the prevalence of alcohol-associated liver disease is 3.5–4.8% in the general population, and 51% of all patients with AUD [5,6].

AUD has a strong biological component, associated with high heritability [7]: 25–30% of persons with an alcoholic parent also develop AUD. The specific mechanisms of heritability are largely unknown and under active investigation, involve hundreds of variants across the genome, and may involve gene influences on systems such as neurotransmission, reward-related learning and memory, and the stress response [8,9]. Persons with AUD have a lower level of clinical response to alcohol and a reduced size of brain neuroanatomy associated with executive functions (dorsolateral prefrontal cortex), risk taking, and decision making (insular cortex) [8]. In fact, negative associations between alcohol intake and brain macrostructure and microstructure are already apparent in individuals consuming an average of only one to two daily units and become stronger as alcohol intake increases [10]. Other neurobiological findings in AUD patients are changes in gene expression over time [11], increased brain activity in the PFC and the anterior thalamus vs. social drinkers [12], higher craving in response to alcohol cues with increased PFC and limbic activity [12], hyper-responsiveness to alcohol-related stimuli [13], and altered striatal dopamine release long-term [14]. With abstinence, some brain volume changes are reversible [15].

A subpopulation, primarily of East Asian descent, demonstrates a flushing reaction similar to the disulfiram-alcohol reaction due to a genetic mutation of chromosome 12q24, which renders ALDH2 nonfunctional in catalyzing acetaldehyde. In vivo measurement of ALDH2 activity in the liver of heterozygotes was found to be 17% of that of homozygotes [16]. No catalytic activity is found in homozygotes.

Patients with AUD are also one of the most underserved populations with psychiatric disorders. In the US, only 7.3% of persons with AUD received treatment of any kind, and only 1.6% were prescribed a medication to treat AUD [17]. After attempting abstinence, approximately 70–80% of patients relapse within a year of treatment [18].

Despite the considerable unmet medical need, there are only four approved drug therapies for AUD with three distinct mechanisms, approved decades ago, all of which have major limitations in efficacy and/or safety: acamprosate [19], naltrexone oral/naltrexone ER (injectable) [19,20], and disulfiram [21,22]. Importantly, acamprosate and naltrexone do not rigorously support abstinence, as both can be taken even as the patient continues to drink alcohol.

For many individuals, abstinence from alcohol is the optimal goal. Disulfiram is an oral treatment for AUD discovered to have an aversive effect when alcohol is imbibed in the 1940s, called the disulfiram–alcohol reaction (DAR) in the literature. Alcohol is metabolized by alcohol dehydrogenase to acetaldehyde, which is then metabolized by aldehyde dehydrogenase to acetic acid and eventually to carbon dioxide and water. Disulfiram is metabolized by CYP-P450 enzymes to many metabolites, including its primary active metabolite, S-methyl N,N-diethylthiolcarbamate (DETC-MeSO), which irreversibly binds and inhibits the ALDH2 enzyme [23,24]. In contrast with disulfiram, which is dosed orally as 250 mg/day, the active metabolite DETC-MeSO has very high potency.

When persons taking disulfiram drink alcohol, the build-up of the toxic substrate acetaldehyde generates a range of adverse effects that can include general discomfort, flushing, drowsiness, headache, blurry vision, sweating, nausea or vomiting, shortness of breath, irregular heartbeat, weakness or fainting, and confusion. The reaction may last for a few hours.

Disulfiram can be effective when used as prescribed [21], but it is an oral drug with documented high nonadherence in clinical trials [22]. Its clinical use is further constrained by hepatotoxicity in a hepatically compromised population, oral dosing requirements, multiple drug–drug interactions, and poor adherence [22]. Adverse effects, including hepatitis, neuropathy [25,26], and rare fatalities, have further limited adoption.

SOPH-110S is a novel ALDH2 inhibitor analog of DETC-MeSO under development for AUD as an injectable formulation. To support its clinical safety and efficacy testing, SOPH-110S was evaluated in a series of nonclinical regulatory-compliant experiments, as appropriate, to assess the mechanism, pharmacokinetics, safety, selectivity, off-target activity, CYP inhibition, and proof-of-concept efficacy in a rat model that included dosing and alcohol challenge followed by analysis of liver ALDH2 inhibition.

## 2. Materials and Methods

**Figure 1:**
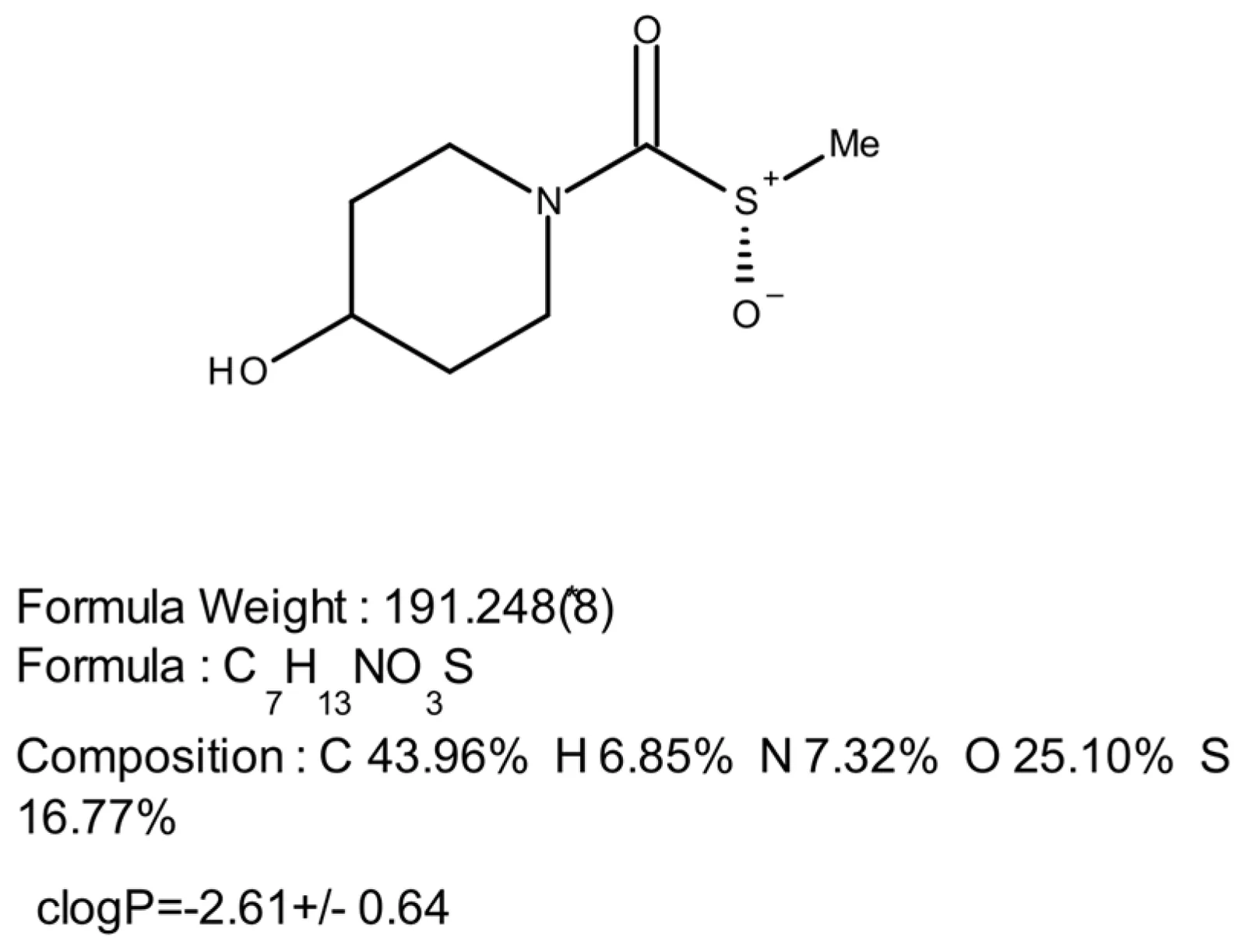
Structure and composition of SOPH-110S. The chirality of SOPH-110S was determined by vibrational circular dichroism (VCD) and confirmed by X-ray structure determination. We have a qualified chiral HPLC method to accurately measure any changes in the %ee. We have stability for up to 12 months. Five degrees below 0 centigrade and below is best for storage.

SOPH-110S is the S enantiomer of a thiocarbamate-S-oxide containing a stereogenic sulfur center (Figure 1). Sulfoxides are conformationally stable at room temperature and, therefore, can be separated into pure enantiomers [25]. The initial racemic synthesis of SOPH-110 was performed at Raybow USA, Brevard, NC, USA, and the chiral separation to produce SOPH-110S was performed under the Raybow GMP protocol (Lotus Separations LLC, Princeton, NJ, USA) resulting in >98% chemical purity and >99% chiral purity.

### 2.1. In Vitro Study Evaluating ALDH2 Inhibition

An in vitro study was performed to evaluate the effects of various compounds and SOPH-110 in both its racemate and S-enantiomer forms on ALDH2 enzyme (Confluence Discovery Technologies, St Louis, MO, USA). Comparison was made with the positive controls known to inhibit ALDH2, i.e., disulfiram and its active metabolite S-methyl-N, N-diethylthiocarbamate sulfoxide (DETC-MeSO).

The assay was conducted close to the optimum pH of the activity peak for ALDH2 (pH 9.0) and at the more physiological pH 6.5.

The compounds, reconstituted in a 0.2% final concentration of DMSO, were added to 96-well plates. An ALDH2 enzyme solution was prepared from a 1 mg/mL stock of active recombinant human ALDH2 protein (rhALDH2; Abcam Inc., Waltham, MA, USA; catalog number: ab87415) to a concentration of 30 ng per 25 μL in base buffer (pH 9.0) (plate A) or a 50 mM sodium phosphate buffer (pH 6.5) (plate B) and added to respective wells. The plates were incubated with gentle shaking at room temperature (RT) for two hours. At the end of the two-hour preincubation, an assay mix consisting of acetaldehyde and NAD+ substrates in base buffer was added to catalyze the reaction for conversion of NAD+ to NADH by ALDH2. The plates were incubated for 30 min for enzymatic conversion, and NADH product formation was quantitated by adding the NAD(P)H-Glo detection reagent (Promega Corporation, Madison, WI, USA; Catalog Number: G9062) to each well. The enzyme reductase used NADH to reduce a pro-luciferin substrate to form luciferin. Luciferin was then quantitated using Ultra-Glo Recombinant Luciferase, and the light signal produced (relative light units: RLU) was proportional to the amount of NADH in the sample. Luminescence readings were obtained using an Analyst HT plate reader (Molecular Devices Corporation, Sunnyvale, CA, USA). The luminescence values were converted to pmol NADH product per well using the standard curves. ALDH2 activity was expressed as the specific activity (SA) given the time of reaction (30 min) and the amount of enzyme (30 ng per well). The activity of the tested compound (i.e., SOPH-110S, DETC-MeSO, or disulfiram) was expressed as nmol of NADH formed per min based on a standard curve with NADH. The measurement was conducted using the NADH-Glo assay. The inhibition of the enzyme by each test compound was determined as a percentage of the negative control, which consisted of 30 ng of rhALDH2 without inhibitors (test articles) as 100%.

The CriterionHost software version 2 (LJL Biosystems, Sunnyvale, CA, USA), Excel^®^ 2010 (Microsoft^®,^ Redmond, WA, USA), and GraFit^®^ version 5 or 7 (Erithacus, East Grinstead, West Sussex, UK) were used for data analysis. Graphs were analyzed using a four-parameter logistic model, and IC_50_s were determined after iterating to the best least-squares fit. The experiment used two (*n* = 2) wells per condition.

### 2.2. Off-Target Binding Assay

SOPH-110S (10 µM) was assessed in a panel of 84 binding targets (including receptors, ion channels, transporters, kinases, and uptake assays). In each experiment, the respective reference compound was tested concurrently with SOPH-110S, and the data were compared with historical values determined at Eurofins (Celle-Lévescault, France).

Compound binding was calculated as the percent inhibition of the binding of a radioactively labeled ligand specific for each target. The compound enzyme inhibition effect was calculated as the percent inhibition of control enzyme activity. Each assay was performed in duplicate, and the mean of the two duplicates was calculated.

### 2.3. Inhibition of CYP Enzymes in Human Liver Microsomes

An in vitro study was conducted to determine the potential of SOPH-110S to cause direct or time-dependent inhibition (TDI) of the major drug-metabolizing liver cytochrome P450 enzymes (CYP1A2, 2B6, 2C8, 2C9, 2C19, 2D6, and 3A) using pooled human liver microsomes (HLMs) and standard probe substrates (Frontage Laboratories Inc., Exton, PA, USA). Direct inhibition was assessed at a range of SOPH-110S concentrations (0.05 to 100 μM) by monitoring CYP-specific metabolite formation and calculating the concentration that caused a 50% reduction in the metabolite formation rate (IC_50_). The potential of SOPH-110S to be a TDI of CYP enzymes was also assessed using the “IC_50_ shift” method, in which the IC_50_ was determined following 30 min pre-incubations of SOPH-110S (0.05 to 100 μM) in pooled HLM with and without NADPH.

### 2.4. Induction of CYP Enzymes in Plateable Human Hepatocytes

An in vitro study was conducted to determine the potential of SOPH-110S to induce CYP1A2, CYP2B6, and CYP3A4 enzymes (Frontage Laboratories Inc., Monmouth Junction, NJ, USA). This study was conducted using primary human hepatocytes from three donors (BEI, BRX, and ZNE) Omeprazole, phenobarbital, and rifampin were used as the positive controls for CYP1A2, CYP2B6, and CYP3A4, respectively. Flumazenil was used as the negative control (no induction). The mRNA levels were determined by real-time qPCR. The baseline values of mRNA levels were obtained from the hepatocytes treated with a vehicle under the same conditions.

### 2.5. hERG Current Amplitude Study

A regulatory-compliant study was conducted to examine the effects of SOPH-110S on the hERG current amplitude recorded under physiological temperatures at a nominal concentration of 50 µM (Nova Research Laboratories, New Orleans, LA, USA). Dofetilide (nominal concentrations of 0.5, 10, and 20 nM) was used as a positive control.

The hERG current was recorded from stably expressing cell lines HEK/hERG, which originated from the human kidney (Nova Research Laboratories, New Orleans, LA, USA). Cells were stored in liquid nitrogen, and a new vial was thawed on the experimental day. Currents were measured using the whole-cell variant of the patch clamp method. Glass pipettes were pulled from borosilicate glass by a horizontal puller (Sutter Instruments, Novato, CA, USA). The internal and external recording solutions were composed of NaCl (137 mM), KCl (4.0 mM), MgCl_2_ (1.0 mM), CaCl_2_ (1.8 mM), HEPES (10.0 mM), and dextrose (11.0 mM), adjusted to a pH of 7.4 with NaOH. The bath temperature was measured by a thermistor placed near the cell under study and was maintained by a thermoelectric device. All experiments were conducted at 37 ± 1 °C. For SOPH-110S-exposed cells, *n* = 5 cells per concentration were tested. For the positive control, *n* = 3, and for the vehicle control, *n* = 3 cells each were tested.

An Axopatch 1-D amplifier (Axon Instruments/Molecular Devices San Jose, CA, USA) was used for whole-cell voltage clamping. Creation of voltage clamp pulses and data acquisition were controlled by a computer running the pClamp software version 10.7 (Molecular Devices San Jose, CA, USA).

### 2.6. Cardiovascular and Body Temperature Effects in Dogs

A regulatory-compliant study was conducted to evaluate cardiovascular effects and body temperature in dogs (Attentive Sciences, Overland Park, KS, USA). Four drug-naïve male beagle dogs, 20 to 23 months old, animals previously implanted with telemetry transmitters (Data Sciences International, St. Paul, MN, USA) for cardiovascular monitoring, were selected from the telemetry colony for use in the study based on a health assessment and confirmation of signal. The dogs received the vehicle or SOPH-110S (0.3, 1, or 3 mg/kg) via subcutaneous injection in a Latin square-dosing regimen, such that each animal received all treatments. There was a minimum off-treatment period of seven days between dose administration. The number of animals used in this study was considered the minimum required to achieve the study objectives, based on regulatory requirements, statistical power, and/or availability of historical data, and was within IACUC-approved guidelines. Only male animals were used because no sex differences were anticipated.

Computer hardware and software supplied by Data Sciences International (DSI, St Paul, MN, USA) were used for radio-telemetry data acquisition. The PhysioTel Digital system with M11R implants was used. These implants, connected to the Dataquest™ OpenART™ Acquisition Interface, provide direct digital signals to the DSI PONEMAH software (version 5.50). ECG and ABP waveforms were sampled at a minimum of 500 Hz. Temperature data was sampled at a minimum of 10 Hz. Data acquired continuously were logged every 60 s.

On each day of dosing, radio-telemetry recording began at ~2 h prior to dosing and continued to ~24 h post-dose.

ECG and blood pressure signals may at times be uninterpretable due to interference caused by an animal’s activity or body positioning. These waveforms were excluded, but all other ECG waveforms that were recognized by the software were included. Qualitative and quantitative assessment of ECG waveforms was performed by an experienced cardiovascular data analyst.

The data captured included heart rate (HR), systolic arterial pressure (SBP), diastolic arterial pressure (DBP), mean arterial pressure (MAP), body temperature (BT), PR interval, RR interval, QRS duration, QT interval, QtcI (individual) interval, and QTcVW (Van de Water’s) interval using the Ponemah Acquisition System.

The following parameters were analyzed post-acquisition on a validated system with EMKA ecgAUTO version 3.5.9 (emka Technologies, Stirling, VA, USA) using a pattern recognition approach: heart rate (HR), systolic arterial pressure (SBP), diastolic arterial pressure (DBP), mean arterial pressure (MAP), body temperature (BT), PR interval, RR interval, QRS interval, QT interval, QTcI (individual), and QTcVW [27]. QTcI interval values were calculated using an individual animal correction factor (IACF). For each animal, the IACF value was calculated from data collected during the vehicle dose day for that animal and applied to each dose level for the same animal. The IACF was generated by fitting a linear regression line to the 1 min means of the QT interval versus heart rate data. QTcI values were calculated using the formula QTc = QT − IACF × (HR − 75) [28].

The data for each required time point was analyzed and assessed on completion of data acquisition, and was the average of 60 min data bins.

Statistical analysis was performed in 5 phases:

Phase I: Four time intervals of 60 min each for hours 1–4 post-dose.
Phase II: Four time intervals of 60 min each for hours 5–8 post-dose.
Phase III: Six time intervals of 60 min each for hours 9–14 post-dose.
Phase IV: Six time intervals of 60 min each for hours 15–20 post-dose.
Phase V: Four time intervals of 60 min each for hours 21–24 post-dose.

Each parameter was analyzed in the analysis phase with a repeated-measure analysis of covariance (RANCOVA). The factors in the model included baseline (BASE) as a covariate, dosing day (PERIOD), ANIMAL, treatment (TRT), time after dose (TIME), and the interaction of the treatment group and time after dose (TRT*TIME). The SAS^®^ (version 9.4) procedure PROC MIXED was used for analysis, and the covariance structure across time was selected by comparing the corrected Akaike’s Information Criterion (AICC) from the following covariance structures: compound symmetric, autoregressive order 1, compound symmetric heterogeneous, and autoregressive heterogeneous order 1. The covariate was defined for each subject as the time-matched baseline observation from the 2 h baseline immediately preceding each dosing day.

Dunnett’s test was used to compare the treated groups with the vehicle control. If the TRT*TIME interaction was statistically significant, Dunnett’s test was conducted individually for each time interval. Otherwise, Dunnett’s test was conducted overall across the pooled time intervals only. All tests were conducted at the 0.05 significance level.

### 2.7. Pharmacokinetics of SOPH-110S Following Intravenous (IV), Subcutaneous (SQ), and Intramuscular (IM) Administration to Beagle Dogs

The PK parameters of SOPH-110S and its primary metabolite SOPH-120 were determined following a single IV (3 mg/kg), SQ (0.3, 1, and 3 mg/kg), or IM (3 mg/kg) administration to three healthy, adult male Beagle dogs (Attentive Sciences, Overland Park, KS, USA). The same animals (3 dogs) were used to assess PK following SC at 1 and 3 mg/kg, and 3 mg/kg IM with a dosing of at least 7 days between doses. A different set of animals (a total of 6 dogs, 3 dogs for each group) were dosed SQ at 0.3 mg/kg and 3 mg/kg IV.

IV injections were administered into a cephalic vein. SQ injections were administered to the intrascapular region using an appropriately sized needle and syringe. IM injections were delivered into the caudal thigh of a hind limb via an appropriately sized needle and syringe.

Blood samples were collected from the jugular vein at 0.083, 0.25, 0.5, 1, 2, 4, 8, and 24 hr post-dosing (IV) or 0.25, 0.5, 1, 2, 4, 8, 24. and 48 h post-dose (SQ and IM). Blood samples were kept on ice until centrifugation at settings of 2000× *g*, 10 min, and 5 °C within 1 h of collection. Plasma was directly transferred to tubes and stored at −70 °C ± 10 °C until analysis using a qualified LC/MS/MS analytical method. The limit of quantitation (LLOQ) was 5 ng/mL for SOPH-110S and 10 ng/mL for SOPH-120.

Individual animal plasma SOPH-110S and SOPH-120 concentration versus time data were imported into Phoenix^®^ WinNonlin^®^ version 8.3.4.295 (Certara; Princeton, NJ, USA) for analysis. The concentration versus time data were analyzed using a noncompartmental extravascular administration model with uniform weighting. Nominal sample collection times were used in estimating the TK parameters. Concentrations that were below the LLOQ were treated as missing for the TK analyses.

The following parameters were estimated whenever possible:

- C_max_: The maximum observed concentration;
- T_max_: The time to reach the peak concentration;
- T_last_: The timepoint with the last measurable concentration;
- AUC_0-t_: The area under the concentration–time curve from time zero to the timepoint with the last measurable concentration, estimated by the linear up/log down trapezoidal method;
- t_1/2_: The terminal half-life.

All parameters were reported to 3 significant figures. T_1/2_ was only reported if the goodness-of-fit parameter, R2, was greater than or equal to 0.9 and the percent of AUC_inf_ extrapolated to time infinity (AUC_%extrap_) was less than or equal to 20%. The data are reported as the means of the calculated values for individual animals.

### 2.8. In Vivo Study of Liver ALDH2 Inhibition in the Presence of Alcohol in Rats

The effects of the SOPH-110S and R enantiomers on liver ALDH2 activity and ALDH2 protein levels following ethanol challenge were evaluated in Sprague-Dawley (SD) rats at Eurofins (New Taipei City, Taiwan). This study was conducted on 8–9-week-old male rats, and various treatments were evaluated, including a negative control (PBS at pH 5.5), a positive control (DETC-MeSO racemate at 6.5 mg/kg), and the two stereoisomeric forms of SOPH-110 (SOPH-110S and SOPH-110R) at 1, 4, and 8 mg/kg. All test materials were injected intravenously (IV) in a volume of 1 mL/rat once daily (QD) from day 1 to day 10 for a total of 10 days (*n* = 6 rats per group). As another positive control, oral disulfiram at 6.5 mg/kg was also given QD during the same study period. Animals then received 20% ethanol at 1 mL/kg (IP) over 8 h following the 10th dose of the test article. Thirty minutes following ethanol challenge, the animals were sacrificed, and the whole liver was harvested, rinsed in cold saline, and snap-frozen to measure ALDH2 activity (Mitochondrial Aldehyde Dehydrogenase (ALDH2) Activity Assay Kit (abcam ab115348)) and ALDH2 protein (using rat aldehyde dehydrogenase 2 family (mitochondrial) ELISA Kit MBS700110; MyBioSource, San Diego, CA, USA). The mean and SEM were calculated. One-way ANOVA and Dunnett’s test were used to determine the statistical significance between the vehicle control and the treated groups. Differences were considered significant at *p* < 0.05 vs. the negative control.

## 3. Results

### 3.1. In Vitro Study Evaluating ALDH2 Inhibition: Results

**Figure 2:**
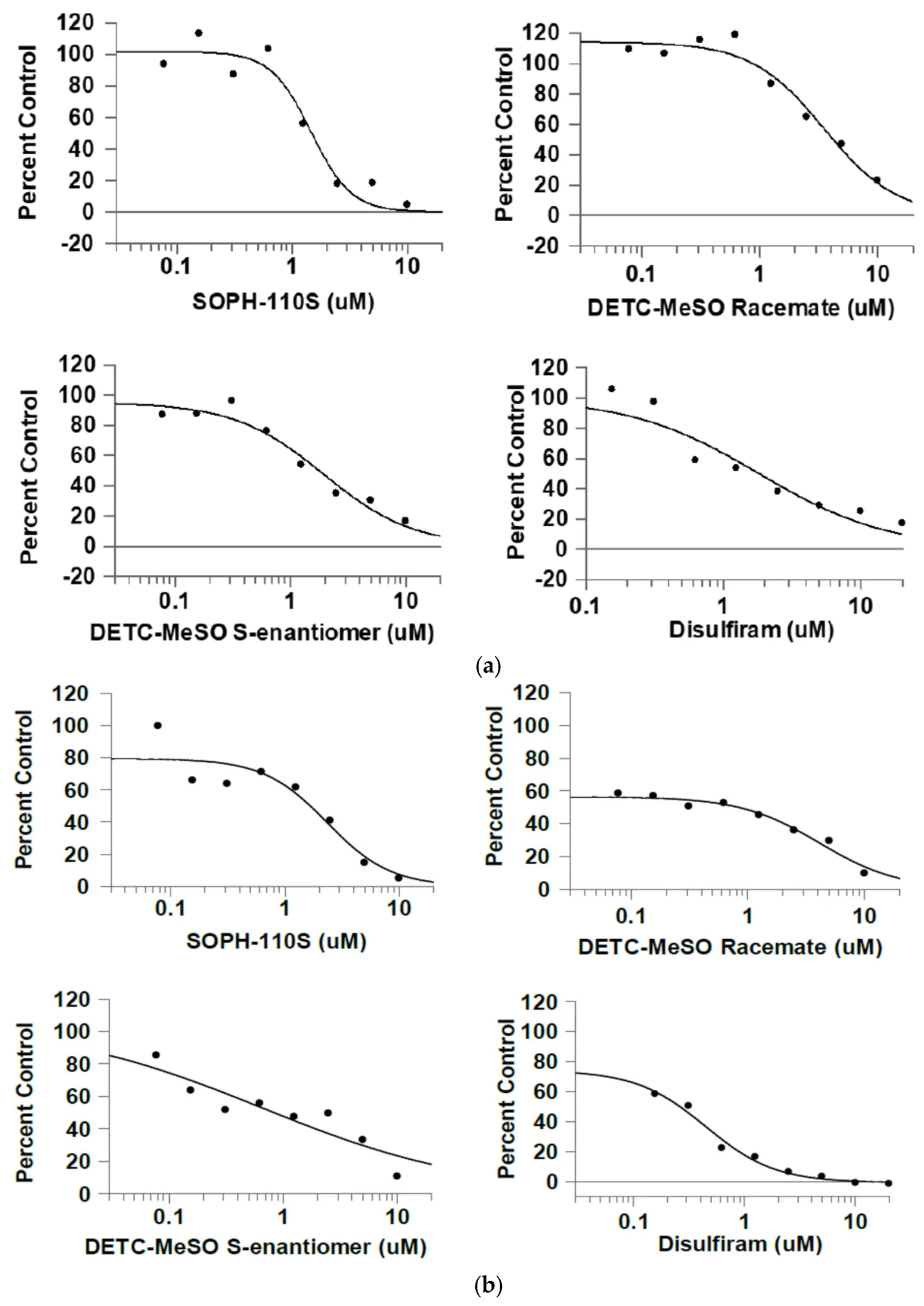
(**a**) Dose-response curves for ALDH2 inhibition by SOPH-110S, DETC-MeSO racemate, DETC-MeSO S enantiomer, and disulfiram at pH 9.0. (**b**) Dose–response curves for ALDH2 inhibition by SOPH-110S, DETC-MeSO racemate, DETC-MeSO S enantiomer, and disulfiram at pH 6.5.

SOPH-110S, DETC-MeSO S-enantiomer, and disulfiram had comparable IC_50_s to each other (1.46 μM, 1.94 μM, and 1.80 μM, respectively) and to the literature (1.45 μM for disulfiram) [16] under standard assay conditions with pH 9.0 (Table 1 and Figure 2a).

The activities were much lower and less reliable using the two-hour preincubation at pH 6.5, followed by an in vitro dehydrogenase assay at pH 7.85 (Table 1). The activity was less than 3% of that in the standard assay (pH 9.0). This was not unexpected based on the known pH activity profile of ALDH2, which peaks at a non-physiological alkalinity near pH 9.0, though it is uncertain whether the loss was due to lower catalytic activity or to protein denaturation. The IC_50_ values, though less accurate, tended to still have comparable relative potencies (Figure 2b), suggesting little relative difference in compound stabilities at a lower pH. The IC_50_s were 2.34 μM, 4.10 μM, 0.657 μM, and 0.493 μM, respectively. The IC_50_s for the DETC-MeSO-S-enantiomer and disulfiram were considerably lower than at pH 9.0.

The IC_50_ values for the compounds SOPH-110S, DETC-MeSO racemate, DETC-MeSO S-enantiomer, and disulfiram at pH 6.5 were considerably higher than at pH 9.0 (Table 1).

### 3.2. Off-Target Binding Assay: Results

Results showing inhibition or stimulation higher than 50% are considered to represent significant effects of the test compound, requiring further investigation (determination of IC_50_ or EC_50_ values from concentration–response curves). Such effects were not observed at any of the receptor targets studied (Table 2).

Results showing an inhibition (or stimulation) between 25% and 50% are indicative of weak to moderate and are within a range where more inter-experimental variability can occur. Three targets were observed with inhibition in the 25% to 50% range (CXCR2 at 32.6%, GABA-gated Cl^−^ channel at 26.4%, and IRK at 25.2%). The significance of this, if any, will be determined in future nonclinical studies.

### 3.3. Inhibition of CYP Enzymes in Human Liver Microsomes: Results

SOPH-110S did not inhibit the activity of CYP1A2, CYP2B6, CYP2C8, CYP2C9, CYP2C19, CYP2D6, and CYP3A enzymes (IC_50_ > 100 μM) (Table 3). Direct positive control inhibitors inhibited CYP activity by 77.2–95% compared with solvent control.

Since SOPH-110S is not a time-dependent inhibitor of the common hepatic drug-metabolizing P450 enzymes, it is not expected to be associated with liver toxicity like disulfiram. Urinary excretion is considered the major excretion pathway of SOPH-110S.

### 3.4. Induction of CYP Enzymes in Plateable Human Hepatocytes: Results

In human hepatocytes from three donors, SOPH-110S at concentrations of up to 100 μM did not produce induction (<2-fold) in CYP1A2 mRNA levels at all concentrations up to 100 μM. The percentage of fold change relative to the positive control (omeprazole) was less than 1% in all preparations at all concentration levels. Under the same experimental conditions, omeprazole (25 μM) produced the expected increase in CYP1A2 mRNA levels (63–332-fold) compared with the vehicle control in all three preparations. The negative control (flumazenil (20 μM)) used in this study produced no change in mRNA levels (1.0–1.5-fold) compared with the vehicle control. Therefore, SOPH-110S is not an inducer of CYP1A2 in human hepatocytes.

Treatment with SOPH-110S did not produce induction (<2-fold) in CYP2B6 mRNA levels at all tested concentrations up to 100 μM in all preparations. The percentage of fold change relative to the positive control (phenobarbital (2 mM)) was less than 3% in all preparations at all concentrations. Under the same experimental conditions, the positive control, phenobarbital, produced the expected increases in CYP2B6 mRNA levels (5–14-fold) compared with the vehicle control in all three preparations. The negative control, flumazenil, produced no change in CYP2B6 mRNA levels (0.8–1-fold) compared with the vehicle control. These results suggest SOPH-110S is not an inducer of CYP2B6 in primary human hepatocytes.

Treatment with SOPH-110S did not produce induction (<2-fold) in CYP3A4 mRNA levels at all concentrations up to 100 μM in all preparations. The percentage of positive control (rifampin) for the fold-change of the CYP3A4 mRNA was less than 0% at all concentration levels in all subjects. Therefore, SOPH-110S is not an inducer of CYP3A4 as well as in primary human hepatocytes.

### 3.5. hERG Current Amplitude Study: Results

SOPH-110S had no effect on hERG current, with the highest concentration tested (50 μM) being associated with a 1.6 ± 1.0% reduction. An IC_50_ value could not be calculated. The response to the positive control, dofetilide (20.9% inhibition at 0.5 nM; 94.0% inhibition at 10 nM; and complete block at 20 nM), and to the vehicle–time control demonstrated proper system performance.

SOPH-110S does not bind to human protein. Allometric scaling was used to predict human exposure SOPH-110S following subcutaneous dosing. Clinically relevant doses are predicted to result in a safety margin of greater than 50-fold for potential hERG interactions and QT/QTc interval prolongation and proarrhythmic potential. However, clinical studies are needed to confirm these results.

### 3.6. Cardiovascular and Body Temperature Effects in Dogs: Results

No biologically relevant effects on measured arterial pressure, ECG, or body temperature parameters were noted.

### 3.7. Pharmacokinetics of SOPH-110S Following Intravenous (IV), Subcutaneous (SQ), and Intramuscular (IM) Administration to Beagle Dogs: Results

#### 3.7.1. SOPH-110S

Following a single SC or IM administration of SOPH-110S, the parent compound (SOPH-110s) was quantifiable for 1 hr post-dose. Following a single IV administration of SOPH-110S, the parent compound (SOPH-110s) was quantifiable for 0.5 hr post-dose. As summarized in Table 4, the T_max_ of SOPH-110S was observed at 0.333 hr following single SC administration of 0.3, 1, or 3 mg/kg of SOPH-110S. Following single IV and IM administration of 3 mg/kg of SOPH-110S, the T_max_ was observed at 0.0833 and 0.250 hr, respectively. Following SC dosing, exposure of SOPH-110S based on C_max_ increased in a less than dose-proportional manner. As the dose increased in a ratio of 1:3.33:10, the C_max_ of SOPH-110S increased in a ratio of 1:1.58:8.78. The AUC_0-t_ increased in a dose-proportional manner in a ratio of 1:2.07:8.23. The terminal phase of the TK profiles was insufficiently characterized to estimate the T_1/2_ of SOPH-110s in all groups.

#### 3.7.2. SOPH-120 (Primary Metabolite)

Following a single IV, SQ, or IM administration of SOPH-110S, SOPH-120 was quantifiable for 4 hr post-dose. Following a single IV, SC, or IM administration of SOPH-110S, the metabolite (SOPH-120) was quantifiable for 4 hr post-dose. As summarized in Table 5, the T_max_ of SOPH-120 was observed at 0.833, 0.667, and 0.799 hr following single SC administration of 0.3, 1, and 3 mg/kg of SOPH-110S, respectively. Following single IV and IM administration of 3 mg/kg of SOPH-120, the T_max_ was observed at 0.250 and 0.417 hr, respectively. Following SC dosing, exposure of SOPH-120 based on the C_max_ increased in a greater than dose-proportional manner. As the dose increased in a ratio of 1:3.33:10, the C_max_ of SOPH-120 increased in a ratio of 1:4.06:16.06.

**Figure 3:**
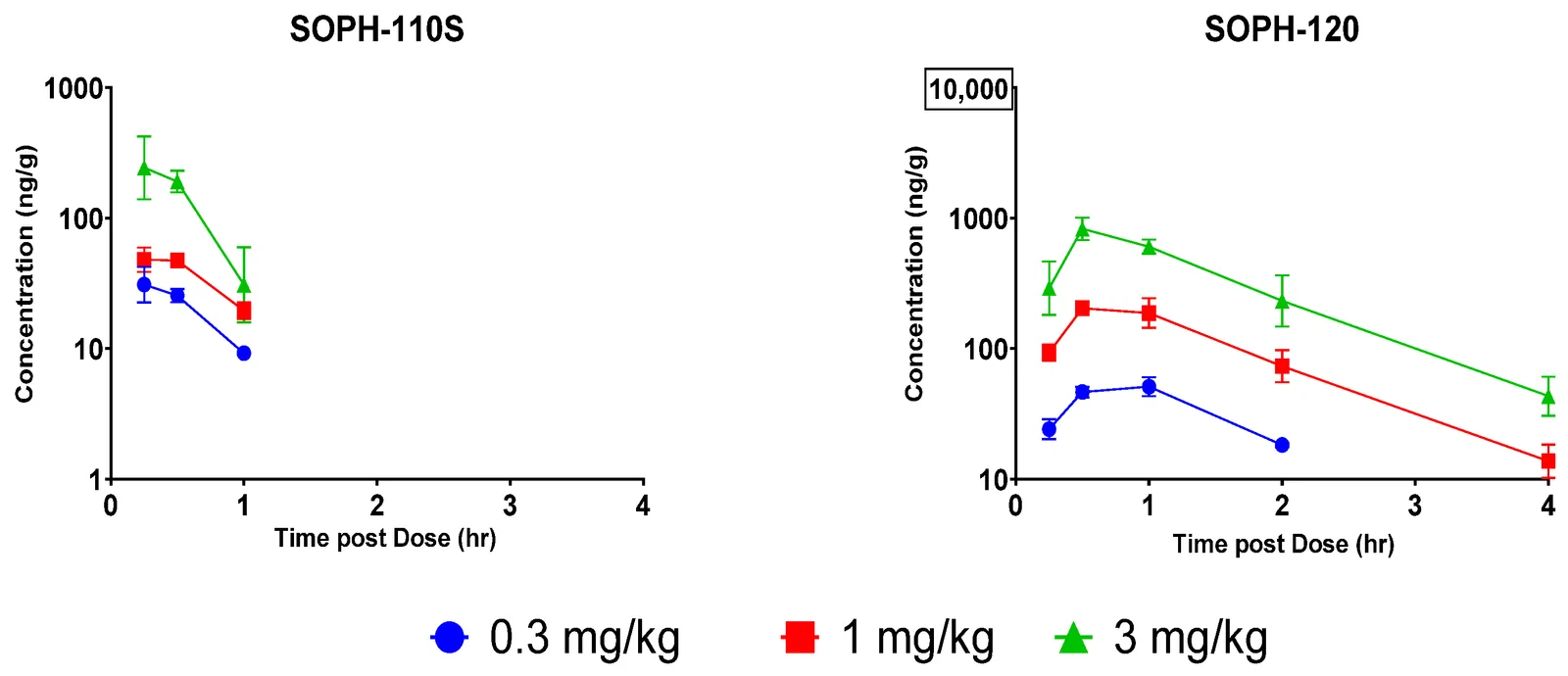
Mean (+SD) concentration vs. time plots for SOPH-110S and SOPH-120 in Beagle dogs following a single SC dose of SOPH-110S at 0.3, 1, or 3 mg/kg (*n* = 3).

The AUC_0-t_ increased in a greater than dose-proportional manner in a ratio of 1:5.04:17.2. The terminal phase of the PK profiles was insufficiently characterized to estimate the T_1/2_ of SOPH-120 following SC administration of 0.3 mg/kg of SOPH-110S. The T_1/2_ was observed at 0.799 and 0.769 hr following a single SC administration of 1 and 3 mg/kg of SOPH-110S, respectively. Similarly, the T_1/2_ was observed at 0.735 and 0.775 hr following a single IM and IV administration of 3 mg/kg of SOPH-110S, respectively. The mean concentration vs. time plots dose comparison for SQ dosing is provided in Figure 3.

### 3.8. In Vivo Study of Liver ALDH2 Inhibition in the Presence of Alcohol in Rats

**Figure 4:**
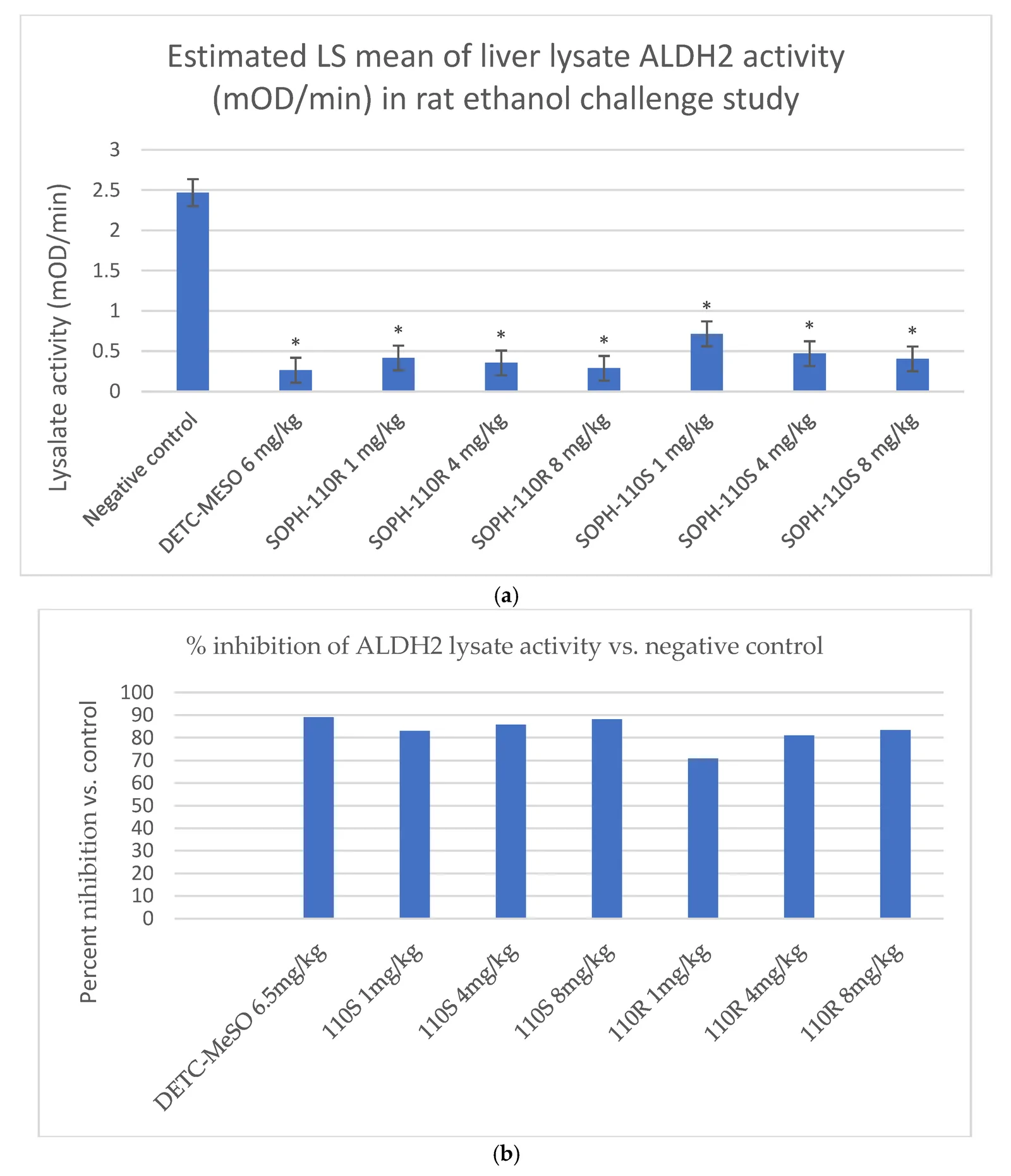
(**a**) Liver lysate ALDH2 activity (mOD/min) measurement in the rat ethanol challenge study. Analysis of variance (ANOVA) with Dunnett’s test was used to determine statistical significance between negative control and treated groups. * indicates significant differences with *p*-value < 0.0001 vs. negative control. Error bars represent the standard error of the LS means. (**b**) Percent of inhibition of ALDH2 lysate activity.

After 10 days’ dosing followed by challenge with alcohol, SOPH-110S was found to be a highly potent, dose-dependent inhibitor of ALDH2, comparable to DETC-MeSO, and numerically more potent than the R-enantiomer of SOPH-110S (Figure 4a,b). Several experiments were conducted using variations on the design described, and there was relatively high variability in negative control values: 0.79, 2.35, 3.73, 3.13, and 2.35 mOD/min (mean: 2.47 mOD/min ± 1.10). Thus, the mean and SD of these values are plotted in Figure 3.

Assay sensitivity was demonstrated in that the positive control, DETC-MeSO, showed significant inhibition of ALDH2 activity compared with the negative control group (Figure 4a; *p* < 0.0001 for all comparisons). SOPH-110S at doses of 1–8 mg/kg produced a dose-dependent 83.0–88.2% reduction in lysate activity (Figure 4b).

## 4. Discussion

### 4.1. Preclinical Data Including Proof of Mechanism

The purpose of this series of experiments was to identify and characterize a novel ALDH2 inhibitor to be brought forward into clinical studies. Both in vitro and in vivo experiments have shown that SOPH-110S is a potent inhibitor of ALDH2. SOPH-110S showed greater inhibitory activity than the R enantiomer, and, thus, SOPH-110S was chosen for further development.

SOPH-110S shows very high selectivity for the target enzyme, with no clinically relevant activity in a standard panel of 84 binding targets, which includes receptors, ion channels, transporters, and other enzymes of potential concern with respect to off-target activity. Although unlikely, it could bind to other clinically relevant targets that may be detected in long-term nonclinical or clinical studies. Future studies should evaluate activity across the ALDH superfamily relative to ALDH2 to further characterize the selectivity of SOPH-110S.

Urinary excretion is considered the major excretion pathway of SOPH-110S.

SOPH-110S showed no hERG liability and no cardiovascular or temperature effects and was detectable in plasma with a relatively short half-life in rats and dogs.

In the in vivo proof of mechanism study, SOPH-110S inhibited ALDH2 after 5 days of dosing at 83–88.2% of the negative control in doses ranging from 1 to 8 mg/kg and was comparable to DETC-MeSO (89.1% inhibition) at all doses, demonstrating that SOPH-110S is a highly potent ALDH2 inhibitor in the presence of alcohol. Future studies should evaluate behavioral responses, such as reduction in alcohol self-administration or relapse in animal models, as well as acetaldehyde levels.

### 4.2. Limitations of Current Treatments for AUD

There has been little innovation in developing drugs to treat AUD in the last several decades. Only three classes of drugs are approved: acamprosate, an N-methyl-D-aspartate (NMDA) receptor modulator; oral naltrexone and long-acting injectable naltrexone (Vivitrol), an opioid receptor antagonist; and disulfiram, an inhibitor of ALDH2.

Acamprosate is approved for the maintenance of abstinence from alcohol in patients with alcohol dependence who are abstinent at treatment initiation. It must be taken three times daily, a schedule that is associated with poor adherence for any oral medication [29]. In the meta-analysis of Maisel et al. (2013) [19], the overall effect size for maintaining abstinence was modest (0.359), and very small for outcomes of heavy drinking (0.072) and reduction in craving (0.034). Naltrexone has a very narrow FDA label, as it is indicated for the treatment of alcohol dependence in patients able to abstain from alcohol in an outpatient setting prior to initiation of treatment. Its effect sizes are even smaller for maintaining abstinence (0.116), reducing heavy drinking (0.189), and reducing craving (0.144) [19,20].

Moreover, both acamprosate and naltrexone can be taken while consuming alcohol and, thus, do not optimally assist the patient in maintaining abstinence.

For large numbers of persons with AUD, long-term abstinence is the necessary and/or desired treatment goal. This group can include individuals with multiple prior relapses, repeated rehabilitation stays, legal mandates (e.g., DWI/DUI), severe psychosocial consequences (such as threatened employment, marital conflict, or parenting failures), and alcohol-related liver disease.

Disulfiram treatment provides a powerful disincentive to continued drinking, via its active metabolite DETC-MeSO [23,24,30]. The clinical effect is to block the reinforcing properties of alcohol and provide an aversive conditioning experience if alcohol is consumed. Patients are educated as to the consequences of drinking while taking disulfiram, and controlled trials have shown that merely the threat of the disulfiram–alcohol reaction (DAR) is sufficient to allow maintenance of abstinence. Petrakis et al. (2005) [21] conducted a large trial in patients with AUD and comorbid psychiatric disorders (*n* = 254), which compared the efficacy of oral naltrexone alone, placebo, disulfiram open treatment, and naltrexone + disulfiram (open treatment). There was a high rate of adherence (82.7%) and a high overall rate of abstinence (69.7%), regardless of the treatment assignment. Naltrexone + disulfiram was not superior to disulfiram alone but was associated with more adverse effects than either treatment alone, which included abdominal pain, nausea, vomiting, numb limbs, pins and needles, irregular heartbeat, and restlessness [21].

However, disulfiram has multiple limitations as a treatment for AUD. Its potential adverse effects in the absence of alcohol include drowsiness, headache, cognitive impairment, nausea, vomiting, abdominal pain, and rashes. It has multiple CYP-P450 interactions and generates multiple metabolites and, thus, has multiple potential drug–drug interactions. Moreover, disulfiram is associated with the rare but serious adverse effects of peripheral neuropathy, believed to be due to one of several metabolites [26] and potentially fatal liver toxicity [25]. This is of major concern in this population, with an approximately 50% rate of comorbid liver disease [5,6].

In addition, the amount of DETC-MeSO generated from a given dose is believed to be highly variable. Since the intensity of the DAR is roughly proportional not only to the amount of total amount of alcohol consumed but also to the total inhibition of ALDH2, it is difficult to carefully titrate the amount of inhibition desired with disulfiram to provide the intended disincentive but maintain a safe level of ALDH2 inhibition in the event of a relapse. In decades past, when the dose of disulfiram was as high as 1 gm, severe DARs have been reported, including respiratory depression, cardiovascular adverse effects, seizures, and death.

### 4.3. Patient Adherence Is Critical to Clinical Outcomes

Adherence to prescribed medication is a major general problem in medicine [31,32]. Poor adherence to treatment is especially problematic in addiction treatment [33]. The nature of the condition is such that craving can drive nonadherence, and brief lapses in oral dosing of disulfiram permit relapse.

Controlled trial data demonstrate that adherence to oral disulfiram can be extremely poor. In the largest controlled trial of disulfiram to date (Fuller et al., 1986 [22]; *n* = 605), there was 80% nonadherence, as demonstrated by urine testing for riboflavin, which was incorporated into the disulfiram tablets. No significant differences over the 1-year period were found on the outcomes of total abstinence, time to first drink, employment, or social stability between the disulfiram group and the two control groups (1 mg of disulfiram and no disulfiram psychosocial treatment). Given the very high rates of nonadherence, this is considered a failed trial. However, there was a highly significant relationship between adherence to dosing and successfully achieved abstinence: among subjects judged adherent, 43% were abstinent vs. only 8% of the nonadherent group [22].

### 4.4. Effects of ALDH2 Inhibition on Psychiatric Symptoms and Craving

In the large trial of patients with AUD and comorbid psychiatric disorders (*n* = 254), disulfiram was found to be superior to placebo and was associated with dramatically improved symptoms of general psychopathology [21]. In addition, the investigational ALDH2 inhibitor ANS-6637 demonstrated dose-dependent improvements in abstinence-related outcomes as well as reduced craving in a small randomized, controlled trial [34]. Selective ALDH2 inhibition suppresses cue-induced reinstatement of alcohol seeking [35], reduces anxiety-like behaviors [36], and attenuates alcohol-induced dopamine release in the nucleus accumbens [37,38]. These findings suggest that ALDH2 inhibition may act through dual pathways, reinforcing abstinence via aversive conditioning while modulating craving-related circuits. We hypothesize that SOPH-110S may also reduce craving as well as reduce heavy drinking and increase rates of abstinence, similar to disulfiram and ANS-6637, and will evaluate these hypotheses in future clinical trials.

### 4.5. Long-Acting Injectables as a Proven Strategy to Improve Adherence and Successful Outcomes

It is Sophrosyne’s intention to develop a depot long-acting formulation of SOPH-110S if it is demonstrated to be safe and well-tolerated in phase 1. An alcohol challenge study will also be conducted in phase 1, which can potentially demonstrate proof of concept in humans after consuming graded amounts of alcohol.

Long-acting injectable formulations have been used successfully for other psychiatric indications associated with poor adherence, such as schizophrenia and opiate use disorder. The use of a long-acting injectable vs. an oral treatment for schizophrenia is associated with improved adherence, fewer 60-day gaps in medication use, and reduced rates of re-hospitalization [39]. In a very large national cohort study in Sweden (*n* = 29,823), long-acting injectable antipsychotics were associated with substantially lower risk of rehospitalization (hazard ratio: 0.78; 95% CI: 0.72–0.84) [40]. In a study of opioid use disorder, the proportion of opioid-negative urine samples was superior for subcutaneous buprenorphine vs. sublingual buprenorphine (35.1% vs. 28.4%) [41].

For AUD medications, the level and quality of supervised dosing are believed to be important to treatment success, especially for disulfiram, since it is an oral drug [42]. Observation or administration of an injectable treatment at longer intervals by a healthcare professional or family member is simple and more convenient than daily supervision of an oral medication, potentially improving outcomes. The use of an LAI can potentially prevent the impulsive discontinuation of an ALDH2 inhibitor, allowing the patient more time to thoughtfully consider the advantages of continued abstinence.

### 4.6. Limitations of the Current Study

This study has several limitations. Nonclinical studies were conducted per FDA guidance and were sufficient for FDA IND approval, but some were conducted with relatively small numbers of animals and in male-only rats. The PK in dogs is short; PK will be evaluated in humans in a planned phase 1 study. Since SOPH-110S is believed to be an irreversible ALDH2 inhibitor, the relationship between pharmacokinetic and pharmacodynamic properties will also be evaluated. Based on the rat PoM study, it is hypothesized that a short PK will still be sufficient to irreversibly inhibit ALDH2. Future studies should also evaluate activity in ALDH2 isoforms, behavioral measures with acetaldehyde kinetics in animal models of AUD, and the feasibility of a long-acting depot injectable with SOPH-110S. Given the known ALD2*2 deficiency in a subpopulation of humans, future evaluation of the safety of SOPH-110S should be explored in subsequent clinical studies.

## 5. Conclusions

Based on data to date, SOPH-110S is a safe, potent in vitro/in vivo ALDH2 inhibitor intended to be developed for AUD treatment as a long-acting injectable. Future studies are planned to explore the development of a depot formulation with a sufficient duration of action to improve adherence in this difficult-to-treat condition. An LAI treatment for AUD, with the options of dosing once weekly, once monthly, or longer intervals, would support sustained abstinence for the many AUD patients who wish to or must maintain abstinence. Clinical and preclinical evidence suggest that an ALDH2 inhibitor may act through the dual pathway of reinforcing abstinence via aversive conditioning while modulating craving-related circuits. An LAI for AUD may offer the opportunity for reduced rates of relapse, better health and quality of life, and generally improved outcomes.

## Figures and Tables

**Table 1:** IC_50_ values for ALDH2 inhibition by SOPH-110S, DETC-MeSO racemate, DETC-MeSO S enantiomer, and disulfiram at pH values of 9.0 and 6.5.

pH	Compound	IC_50_ (µm)	Standard Error (SE)	Slope	Max.	Min.
9.0	SOPH-110S	1.46	0.27	2.50	102	*
	DETC-MeSO Racemate	3.50	0.59	1.41	114	*
	DETC-MeSO S-enantiomer	1.94	0.50	1.12	95.1	*
	Disulfiram	1.80	0.44	0.92	**	*
6.5	SOPH-110S	2.34	0.796	1.56	79.4	*
	DETC-MeSO Racemate	4.10	0.630	1.28	56.4	*
	DETC-MeSO S-enantiomer	0.657	2.31	0.462	106	*
	Disulfiram	0.463	0.151	1.49	71.7	*

* Minimum set to 0. ** 0 to 100% fit.

**Table 2:** Mean inhibition by SOPH-110S (10 µM) in in vitro off-target assays.

Target	% Inhibition	Target	% Inhibition	Target	% Inhibition
A_1_	−1.8	CXCR2 (IL-8B)	32.6	GR	0.3
A_2A_	−0.3	CCR1	0.2	Estrogen ER alpha	9.5
α_1A_	3.0	H_1_	−4.9	PR	8.7
α_1B_	−3.8	H_2_	−5.6	AR	−3.3
α_1D_	6.8	CysLT_1_ (LTD_4_)	−7.0	V_1a_	−1.9
α2_A_	−3.5	MC_1_	8.0	Ca^2+^ channel (L, dihydropyridine site)	−6.0
α2_B_	−4.0	MC_4_	3.8	Ca^2+^ channel (L, diltiazem site)	14.3
β_1_	3.6	MAO-A	9.5	Ca^2+^ channel (L, verapamil site)	4.5
β_2_	−5.1	M_1_	2.0	Ca^2+^ channel (N)	−3.7
AT_1_	−13.6	M2	−5.7	Potassium channel hERG	16.9
BZD	−13.7	M3	4.8	Kv channel	0.2
Cl- channel (GABA-gated)	26.4	M4	15.1	Na^+^ channel (site 2)	−2.9
B_2_	−2.6	NK1	−19.9	Adenosine transporter	0.6
CB_1_	−6.7	Y1	−6.8	Norepinephrine transporter	0.6
CB_2_	4.6	N neuronal α4β2	−7.6	Dopamine transporter	12.2
CCK_1_	22.8	N muscle-type	−0.6	GABA transporter	2.71
CCK_2_	−13.3	δ (DOP)	4.5	5-HT transporter	−11.1
D_1_	−5.5	Kappa (KOP	6.9	COX1	11.8
D_2S_	−3.2	μ (MOP)	2.1	COX2	−11.3
D_2L_	−4.4	PPARγ	−2.1	PDE3A	8.6
ET_A_	0.0	PCP	−2.5	PDE4D2	−2.6
GABA_A1_	−7.3	RARα	−1.6	ACE	−6.4
GABA_B(1b)_	−7.4	5-HT_1A_	3.4	Cathepsin G	1.3
AMPA	−6.3	5-HT_1B_	23.8	IRK	25.2
Kainate	−4.8	5-HT_2A_	−8.3	Lck kinase	−20.7
NMDA	−5.8	5-HT_2B_	−25.5	PKCa	4.0
Glycine (strychnine-sensitive)	−3.9	5-HT_2C_	−1.0	acetylcholinesterase	5.0
Glycine (strychnine-insensitive)	−8.1	5-HT_3_	5.6		
ATPase	0.6	MAO-b	−5.6		

**Table 3:** Summary of the direct and time-dependent inhibition of CYP enzymes by SOPH-110S.

CYP	Substrate	Direct Inhibition IC_50_ (μM)	Time-Dependent Inhibition IC_50_ (μM)		
			No NADPH Pre-Incubation ^1^	With NADPH Pre-Incubation ^2^	Fold Δ in IC_50_
1A2	Phenacetin	>100	>100	>100	None
2B6	Bupropion	>100	>100	>100	None
2C8	Amodiaquine	>100	>100	>100	None
2C9	Diclofenac	>100	>100	>100	None
2C19	S-mephenytoin	>100	>100	>100	None
2D6	Dextromethorphan	>100	>100	>100	None
3A	Midazolam	>100	>100	>100	None
3A	Testosterone	>100	>100	>100	None

^1^ Microsomes, buffer, and SOPH-110S pre-incubated for 30 min prior to initiation of reaction with NADPH and probe substrate. ^2^ Microsomes, buffer, and SOPH-110S pre-incubated for 30 min with NADPH prior to initiation of reaction with probe substrate. The fold change observed does not meet the threshold of significance for a TDI effect (typically when an IC_50_ shift ≥ 2.0).

**Table 4:** Mean and SD PK parameters of SOPH-110S following a single subcutaneous (SC; 0.3, 1, or 3 mg/kg), intramuscular (IM; 3.0 mg/kg), or intravenous (IV, 3 mg/kg) dose of SOPH-110S to male Beagle dogs.

Route	Dose (mg/kg)	Animal ID	T_max_ (h)	C_max_ (ng/mL)	T_last_ (h)	AUC_0-t_ (h.ng/mL)	t_1/2_ (hr)
SC	0.3	**Mean**	0.333	32.2	0.833	16.4	NE
		**SD**	0.144	8.51	0.289	4.19	N/A
SC	1.0	**Mean**	0.333	50.9	1.00	33.9	NE
		**SD**	0.144	8.23	0.00	3.05	N/A
SC	3.0	**Mean**	0.333	283	1.00	135	NE
		**SD**	0.144	108	0.00	30.8	N/A
IM	3.0	**Mean**	0.250	566	0.833	170	NE
		**SD**	0.00	282	0.289	111	N/A
IV	3.0	**Mean**	0.0833	768	0.50	98.8	NE
		**SD**	0.000	124	0.00	15.2	N/A

NE: not estimated (insufficient characterization of the terminal phase to estimate this parameter); N/A: not applicable (N ≤ 2).

**Table 5:** Mean and SD PK parameters of SOPH-120 following a single subcutaneous (SC; 0.3, 1, or 3 mg/kg), intramuscular (IM; 3.0 mg/kg), or intravenous (IV; 3 mg/kg) dose of SOPH-110S to male Beagle dogs.

Route	Dose (mg/kg)	Animal ID	T_max_ (h)	C_max_ (ng/mL)	T_last_ (h)	AUC_0-t_ (h.ng/mL)	t_1/2_ (hr)
SC	0.3	**Mean**	0.833	52.9	2.00	68.6	NE
		**SD**	0.289	8.36	0.00	4.51	N/A
SC	1.0	**Mean**	0.667	215	4.00	346	0.799
		**SD**	0.289	30.8	0.00	70	N/A
SC	3.0	**Mean**	0.667	850	4.00	1180	0.769
		**SD**	0.289	137	0.00	165	N/A
IM	3.0	**Mean**	0.417	1460	4.00	1620	0.735
		**SD**	0.144	85	0.00	396	0.0508
IV	3.0	**Mean**	0.25	1510	4.00	1340	0.775
		**SD**	0.00	219	0.00	174	0.0551

NE: not estimated (insufficient characterization of the terminal phase to estimate this parameter); N/A: not applicable (N ≤ 2).

## Data Availability

The datasets presented in this article are not readily available because they were extracted from numerous large and proprietary study reports. Requests to access the datasets should be directed to the corresponding author.

## Abbreviations

The following abbreviations were used in this manuscript:

ALDH2	Aldehyde dehydrogenase 2
AUD	Alcohol use disorder
BT	Body temperature
CYP	Cytochrome P450
DAR	Disulfiram alcohol reaction
DBP	Diastolic blood pressure
DETC-MeSO	S-methyl-N, N-diethylthiocarbamate sulfoxide
hERG	Human ether-à-go-go-related gene
HR	Heart rate
IC_50_	Half-maximal inhibitory concentration
IM	Intramuscular
IV	Intravenous
MAP	Mean arterial pressure
mM	Millimolar
μM	Micromolar
mOD	Rate of change in optical density
mRNA	Messenger ribonucleic acid
NAD, NADH	Nicotinamide adenine dinucleotide (hydride)
nM	Nanomolar
Pk	Pharmacokinetics
pM	Picomolar
SBP	Systolic blood pressure
SD	Standard deviation
SQ	Subcutaneous
WHO	World Health Organization
